# Emerging Role of MicroRNAs in the Therapeutic Response in Cervical Cancer: A Systematic Review

**DOI:** 10.3389/fonc.2022.847974

**Published:** 2022-06-07

**Authors:** Gloria Ravegnini, Francesca Gorini, Giulia Dondi, Marco Tesei, Eugenia De Crescenzo, Alessio G. Morganti, Patrizia Hrelia, Pierandrea De Iaco, Sabrina Angelini, Anna Myriam Perrone

**Affiliations:** ^1^Department of Pharmacy and Biotechnology (FABIT), University of Bologna, Bologna, Italy; ^2^Division of Oncologic Gynecology, IRCCS Azienda Ospedaliero-Universitaria di Bologna, Bologna, Italy; ^3^Department of Medical and Surgical Sciences (DIMEC), University of Bologna, Bologna, Italy; ^4^Department of Experimental, Diagnostic and Specialty Medicine (DIMES), University of Bologna, Bologna, Italy

**Keywords:** cervical cancer, miRNAs, radiotherapy, chemotherapy, HPV, therapeutic response

## Abstract

Cervical cancer is a common female cancer, with nearly 600,000 cases and more than 300,000 deaths worldwide every year. From a clinical point of view, surgery plays a key role in early cancer management, whereas advanced stages are treated with chemotherapy and/or radiation as adjuvant therapies. Nevertheless, predicting the degree of cancer response to chemotherapy or radiation therapy at diagnosis in order to personalize the clinical approach represents the biggest challenge in locally advanced cancers. The feasibility of such predictive models has been repeatedly assessed using histopathological factors, imaging and nuclear methods, tissue and fluid scans, however with poor results. In this context, the identification of novel potential biomarkers remains an unmet clinical need, and microRNAs (miRNAs) represent an interesting opportunity. With this in mind, the aim of this systematic review was to map the current literature on tumor and circulating miRNAs identified as significantly associated with the therapeutic response in cervical cancer; finally, a perspective point of view sheds light on the challenges ahead in this tumor.

**Systematic Review Registration:**

PROSPERO (CRD42021277980).

## Introduction

### Cervical Cancer

Cervical cancer (CC) is the cancer with the greatest incidence in developing countries, with over 300,000 deaths worldwide each year (1). It recognizes an etiology in most cases associated with infection and cell integration of Human Papilloma Virus (HPV) (2). Given that, it is quite clear that the disease is largely preventable, and about 90% of the CC cases occur in low-income and middle-income countries that do not provide widely planned screening or HPV vaccination programs (2, 3). The most common histological type of CC is squamous cell carcinoma (SCC) with a percentage ranging between 75% and 90%, while the remaining portion is represented by adenocarcinomas (4, 5) and few rare types.

From a clinical point of view, surgery plays an important role in early cancer management, whereas advanced stages are treated with chemotherapy and radiation as adjuvant therapies to eliminate the disease (1, 6). In some cases, neoadjuvant chemotherapy (NAC) can also be employed to reduce tumor mass before surgical approaches (7). However, when cancer does not respond to concomitant therapies, salvage surgery is carried out with demolition procedures that in most cases require emptying the pelvis and definitive urostomies and colostomies (8, 9). Predicting the degree of cancer response to chemotherapy/radiation therapy from the diagnosis to personalize the clinical approach represents the biggest challenge in locally advanced cancers. The feasibility of such predictive models has been repeatedly assessed using histopathological factors, imaging and nuclear methods (10, 11), tissue and fluid scans, however with poor results. In this context, the identification of novel potential biomarkers remains an unmet clinical need.

### MicroRNAs

MicroRNAs (miRNAs) are short non-coding RNAs that are able to regulate the expression of several target genes by complementary binding to specific seed sequences (12–14). Considering that the seed sequence can be formed by 2–8 nucleotides and that the complementarity may also be imperfect, a single miRNA may potentially modulate hundreds of mRNAs (15). Physiologically, mRNAs and miRNAs work in concert and basically control every biological process. However, in pathological conditions, miRNA levels can be deregulated both as a cause and as a consequence of the disease itself, promoting altered conditions including cancer. Over time, the role of miRNAs has been progressively clarified and, even if some aspects have not been completely understood, miRNAs may represent biomarkers or surrogate markers of diagnosis and prognosis (16). Moreover, it has been widely reported that miRNAs can affect the response to a variety of therapeutic treatments, and their expression can be associated with chemosensitivity and radiosensitivity (17, 18).

In recent years, compelling evidence showed that, besides tumor tissue, miRNAs are detectable in every type of body fluid, including but not limited to blood, saliva, tears, and urine. It has been supposed that cancer cells, as well as normal cells, release circulating miRNAs as messengers to send specific messages and communicate with distant cells.

Since their discovery, an increasing number of research groups have demonstrated the involvement of miRNAs in cancer (12, 15), and CC has not been excluded (19, 20). Indeed, many studies have identified different miRNAs as potential diagnostic and prognostic biomarkers in CC. However, in most papers, deregulation was detected when comparing tumor with a normal counterpart or healthy tissue. On the other hand, reports evaluating miRNA expression in relation to pharmacological response are limited and with small consensus. Given these premises, the aim of this review is to provide an overview of the current literature on tumor tissue and circulating miRNAs that are identified to be significantly associated with the therapeutic response in CC.

## Methods

### Systematic Review of Studies Investigating Tumor Tissue and Circulating miRNAs in Therapeutic Response in Cervical Cancer Patients

For this purpose, we systematically searched for papers analyzing the expression of tissue and circulating miRNAs in CC in relation to the therapeutic response.

**Figure 1:**
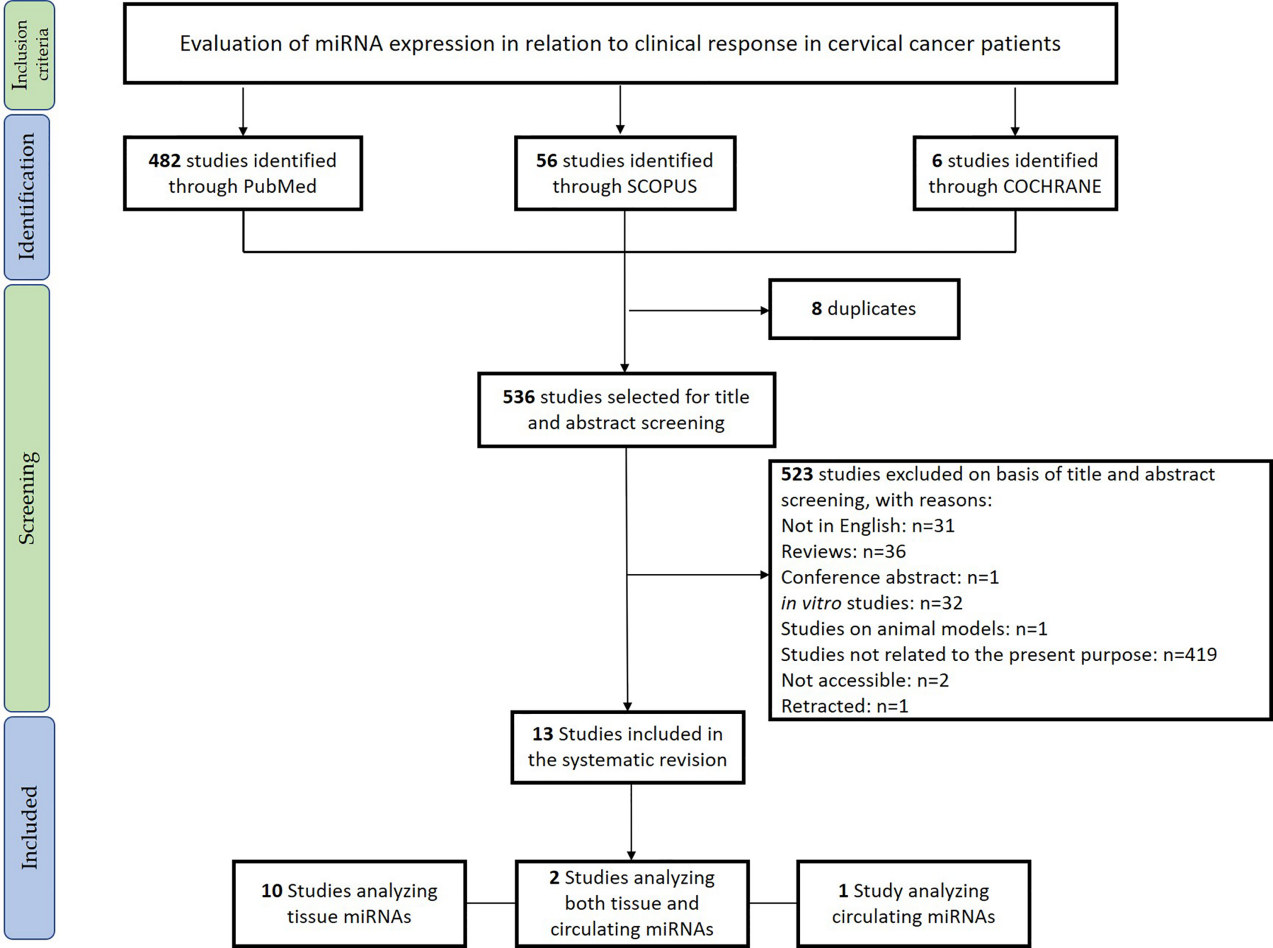
Preferred Reporting Items for Systematic Reviews and Meta-Analyses (PRISMA) flowchart for the studies included in the systematic review.

The systematic review was conducted in accordance with the Preferred Reporting Items for Systematic Reviews and Meta-Analyses (PRISMA) Statement principles (21, 22). The research question was “Can miRNAs be used as biomarkers to monitor therapeutic response in cervical cancer?,” and it was determined using the PICOS process (Population, Intervention, Comparison, Outcomes, Study design) (23). The protocol was registered in the PROSPERO international register on October 10, 2021 (CRD42021277980). PubMed, Cochrane library, and Scopus databases were systematically searched for original articles analyzing the miRNAs associated with drug response in cervical cancer (last updated search December 1, 2021). The papers included in this revision are summarized in **Table 1**. Relevant studies were selected using the Boolean combination of the following key terms: “treatment AND response” OR “therapy AND response” AND “cervical AND cancer” AND “circulating microRNA” OR “microRNA OR miRNA.” Additionally, the reference lists of reviews, meta-analyses, and all original studies were hand-searched to acquire further relevant studies missed from the initial electronic search (**Figure 1**).

**Table 1:** Studies included in the systematic review.

Author, year, [ref]	Aim of the study	Number of patients	Stage	HPV genotype	Therapy	Biological matrix	Technique/s used	Validation of the results	Most important findings	Response definition
***miRNA expression in cervical cancer and chemotherapy + radiotherapy***										
Fekete et al., 2020 (24)	To identify predictive miRNAs in platinum-treated SCC	n = 94 SCCs (from GDC data portal): n = 16 non-responders vs. n = 78 responders	Unknown	NE	Platinum-based chemo	T	MiRNA-seq	\	↑ let-7g, miR-150, miR-155, miR-342, miR-378a, miR-378c, miR-378d-2, miR-502, miR-5586, miR-7702 in responders vs. in non-responders	Response defined based on disease progression at 18 months.
Liu et al., 2018 (25)	To define the role of miR-492 in SCC	-discovery cohort (n = 6: n = 3 sensitive vs. n = 3 resistant pts) - validation cohort (n = 104 CCs: n = 78 sensitive vs. n = 26 resistant pts)	n = 57: stage IIb, n = 47: stage IIIb	NE	Platinum-based chemo + radio	T	RT-PCR based approach (TaqMan Array and assay)	Y - In pts - In cell models - In animal models	↑ miR-492 in sensitive vs. resistant pts ↑ associated with LNM	Resistance defined after 12 months after completion of first-line therapy
Pedroza-Torres et al., 2016 (26)	To identify a set of miRNAs to predict the response in locally advanced CC pts receiving radiation and chemotherapy treatment.	n = 41 CCs: -discovery cohort (n = 10: n = 5 NR vs. n = 5 CR) - validation cohort (n = 31: n = 15 NR vs. n = 16 CR)	IIb/IIIb	E	Platinum-based chemo + radio	T	RT-PCR based approach (miScript miRNA PCR Array and Taqman assay)	Y In pts	↓ miR-100-5p, miR-125a-5p, miR-125b-5p, miR-200a-5p, miR-342 in NR vs. CR.↑ miR-31-3p, miR-3676 in NR vs CR. 7 miRNAs signature associated with DFS	Response evaluated through the RECIST criteria and computed axial tomography scans
Fan et al., 2016 (27)	To study the relationship between miR-125a and resistance in CC	n = 43 CCs: n = 23 responders vs. n = 20 non-responders	n = 21: stage I/II, n = 22: stage III/IV	NE	Taxol and platinum-based chemo	T	Microarray and RT-PCR	Y - In pts - In cell models - animal models	↓ miR-125a in non-responders vs. responders ↓ miR-125a: ↓ PFS, OS, Response Rate	Response defined according the RECIST criteria
Chen et al., 2014 (28)	To clarify the role of miR-181a in regulating the chemoresistance of CC	n = 18 SCCs: n = 7 resistant vs. n = 11 sensitive pts	n = 18: stage IIIB	NE	Platinum-based chemo + radio	T	RT-PCR	Y - In cell models - In animal models	↑ miR-181a in resistant vs. in sensitive pts	Resistance defined as described by Ke et al. (29)
Ke et al., 2013 (29)	To define the roles of miR-181a in determining sensitivity of CC to radiation therapy	n = 18 SCCs: n = 7 resistant vs. n = 11 sensitive pts	n = 18: stage IIIB	NE	Platinum-based chemo + radio	T	Microarray and RT-PCR	Y - In the same cohort - In cell models - In animal models	↑ miR-181a in resistant vs. in sensitive pts	Resistance defined based on histological finding of residual tumor cells in the cervical biopsies sampled 6 months after completion of radiotherapy
***miRNAs expression in cervical cancer and radiotherapy***										
Wei et al., 2020 (30)	To understand the role of miR-411 in radiotherapy response	n = 141 CCs: n = 92 responders vs. 49 non responders	n = 55: stage I, n = 62: stage II, n = 24: stage III	E	Radio	T/PB	RT-PCR	Y In cell models	↑ miR-411 in responders vs. in non-responders in both tissue and blood ↑ miR-411 associated with higher OS and PFS	Efficacy defined according to the RECIST criteria
Gao et al., 2019 (31)	To investigate the biological role of GAS5 in the radiosensitivity	n = 20 CCs: n = 9 resistant vs. n = 11 sensitive pts	IIb to IVb	NE	Radio	T	RT-PCR	Y -In cell models -In animal models	↑ miR-106b in resistant vs. in sensitive pts	Response defined according to the histological results of residual tumor cells in cervical biopsy samples 6 months after completion of radiotherapy
Wei et al., 2017 (32)	To evaluate miR-145 in CCs and investigate its biomarker potential	n = 120 CCs: n = 68 CR vs. n = 52 IR	n = 77: stage I–II; n = 43: stage III	E	Radio	P	RT-PCR	N	↑ miR-145 in CR than in IR pts	Response defined at 6 months after radical radiotherapy
Liu et al., 2015 (33)	To examine the role of miR-18a in regulating the radiosensitivity of CC	n = 48 CCs: n = 20 resistant vs. n = 28 sensitive pts	n = 34: stage I–IIb, n = 14: stage IIIa–Iv	NE	Radio	T	RT-PCR	Y In cell models	↑ miR-18a in sensitive vs. resistant	Response defined at 6 months after radical radiotherapy
Song et al., 2015 (34)	To explore the association between miR-375 and radioresistance in HR-HPV (+) CC	n = 22 CCs: n = 13 resistant vs. n = 9 sensitive pts	Ia/Ia2	E	Radio	T/S	RT-PCR	Y In cell models	↓ miR-375 in resistant vs. in sensitive	Resistance assessed by histological examination of residual tumor tissues 6 months after completion of radiotherapy
***miRNA expression in cervical cancer and neoadjuvant treatment***										
Chen et al., 2014 (35)	To investigate the role of miR-143 expression in cervical SCC	n = 24 SCCs with and without NAC therapy (from a total cohort of 77 CCs and 20 normal cervix tissue)	n = 13. Stage Ib2, n = 9: stage IIa, n = 2: stage IIb	E	Taxol and platinum-based chemo	T	RT-PCR	/	↑ miR-143 after NAC	According to the WHO criteria *
Sun et al., 2013 (36)	To examine the hypothesis that NAC improves prognosis and outcomes after LRH	n = 21 CCs: n = 10 LHR vs. n = 11 NAC+LHR	IIb	E	Taxol and platinum-based chemo	T	RT-PCR	Y In cell models	↑ miR-34a, miR-605 in NAC treated vs. NAC non-treated treated pts	Response defined according to the WHO criteria *

*Complete remission (tumor completely disappeared); partial remission (tumor size decreased more than 50%); stable or no change (tumor size increased or decreased no more than 25%), progression (new lesions or tumor size increased more than 25% during the treatment).

CC, cervical cancer; chemo, chemotherapy; CR, complete response; E, evaluated; HPV, human papillomavirus; HR, high-risk; IR, incomplete response; LRH, laparoscopical radical hysterectomy; NAC, neoadjuvant chemotherapy; NR, no response; Y, yes; N, no; NE, not evaluated; P, plasma; PB, peripheral blood; pts, patients; S, serum; SCC, squamous cell carcinoma; radio, radiotherapy; T, tissue; ↑, higher; ↓, lower; +, positive.

Eligible studies were required to meet the following inclusion criteria: studies evaluating tissue and circulating miRNAs in relation to the therapeutic response in CC. Exclusion criteria were: 1) meta-analyses, reviews, and editorials; 2) non-human studies; 3) *in vitro* studies; 4) non-English articles.

After removing duplicate studies, two investigators (GR and FG) independently checked titles and abstracts of the retrieved articles and judged their eligibility. Then, the entire text of potentially eligible studies was evaluated to assess the appropriateness of inclusion in this systematic review. The same two authors independently extracted the following data from the selected papers: 1) first author, publication year, and aim; 2) sample size; 3) CC stage; 4) evaluation of HPV genotype; 5) type of therapy; 6) type of biological material used for the analysis (tissue/blood/plasma/serum); 7) techniques used and (8) validations; 9) main findings of the report. Disagreements were resolved by discussion with a third reviewer (AMP). Results of the review were discussed with all authors for multidisciplinary topics.

The methodological quality of the cohort studies was evaluated by two investigators (GR and FG) based on an adapted “Quality Assessment Tool for Observational Cohort and Cross-Sectional Studies” proposed by the NIH (37).

## Results

**Figure 2:**
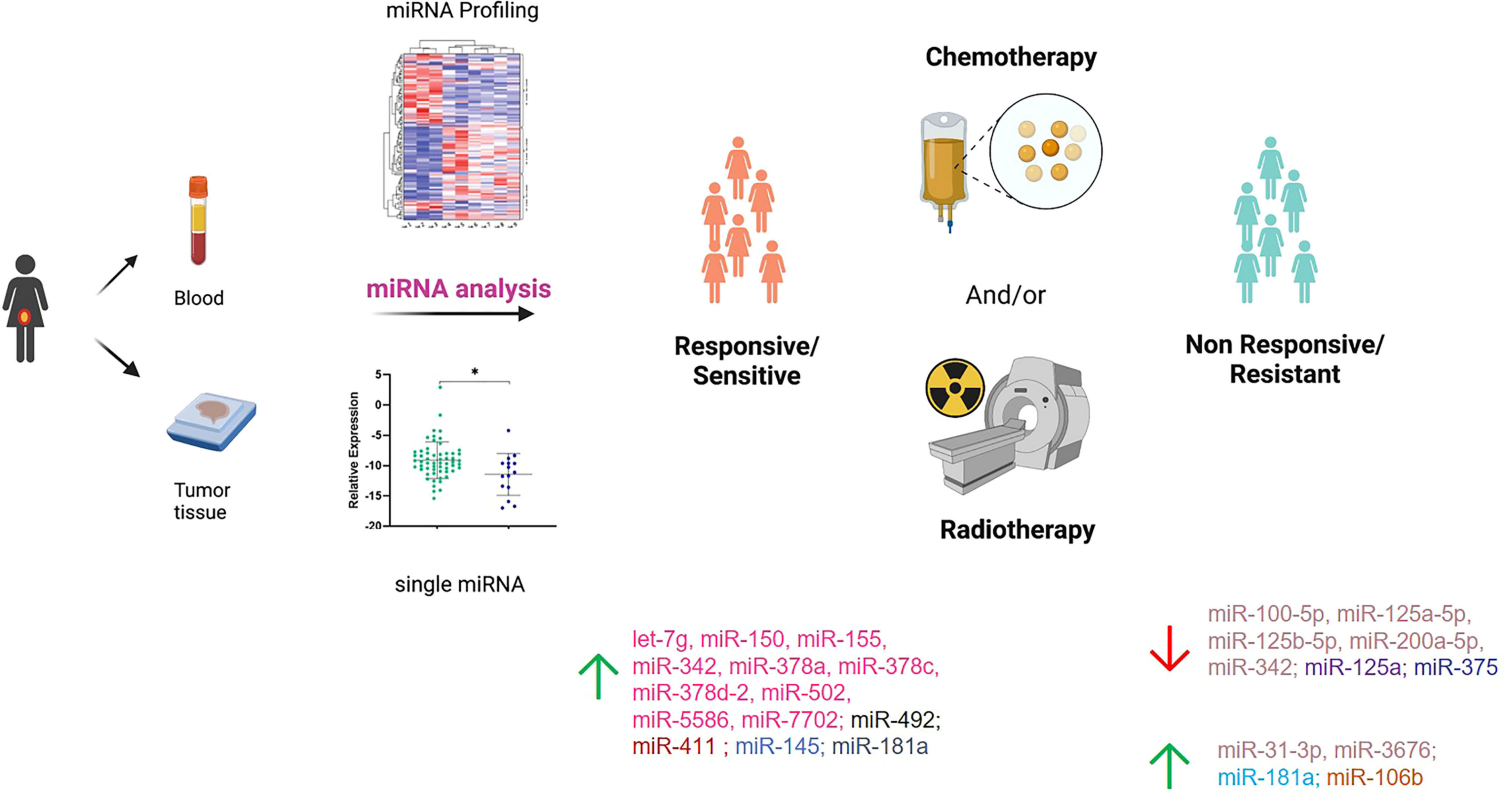
Summary of the miRNAs investigated in responsive/sensitive vs. non-responsive/resistant cervical cancer patients. miRNAs highlighted with the same color derive from the same paper. Created in part with BioRender.com.

We included in the final review a total of 13 papers. The list of papers is reported in **Table 1**, while **Table 2** reports all of the miRNAs evaluated with the suggested targets. The majority of the studies analyzed miRNAs in tumor tissue specimens, a small portion (n = 2) investigated tissue and circulating miRNAs on the same study cohorts (30, 34), and one analyzed plasmatic miRNAs only (32). Overall, most of the studies were based on a comparison between treatment-resistant and non-resistant CC patients (**Figure 2**); in particular, 6 of the 13 papers evaluated miRNAs in patients treated with chemotherapy and radiotherapy, 5 in radioresistant or non-resistant CC patients, and 2 analyzed miRNAs in association with NAC. With regard to the studies performed in tumor tissue, the analysis was carried out starting from tissue preserved in formalin-fixed paraffin-embedded (FFPE) or frozen tumor stored at -80°C until use and collected before any type of treatment. Quality evaluation is summarized in **Table 3**. No study was rated as having good quality; however, four of the 14 criteria were non-applicable to these studies, while one was applicable to one study only. The most common biases were the absence of sample size justification and adjustment of statistical analysis for potential confounding variables.

**Table 2:** Summary of the miRNAs analyzed.

Tissue miRNAs		
miRNA ID	Reference describing the miRNA	Potential targets of miRNAs
let-7g	Fekete et al. (24)	/
miR-100-5p	Pedroza-Torres et al. (26)	/
miR-106b	Gao et al. (31)	IER3, GAS5
miR-125a	Fan et al. (27)	STAT3, ERBB2 and ERBB3, VEGF-A
miR-125a-5p	Pedroza-Torres et al. (26)	STAT3
miR-125b-5p	Pedroza-Torres et al. (26)	BAK1
miR-143	Chen et al. (35)	BCL2, KRAS, MACC1
miR-150	Fekete et al. (24)	/
miR-155	Fekete et al. (24)	/
miR-18a	Liu et al. (33)	ATM, PARP
miR-181a	Chen et al. (28) Ke et al. (29)	PRKCD, RalA
miR-200a-5p	Pedroza-Torres et al. (26)	NRAS, NR4A1, MAPK8, PDGFA, TCF4, DKK2, PSEN1, FZD1, NOTCH2, NOTCH4
miR-31-3p	Pedroza-Torres et al. (26)	
miR-34a	Sun et al. (36)	E2F1
miR-342	Pedroza-Torres et al. (26) Fekete et al. (24)	/
miR-3676	Pedroza-Torres et al. (26)	/
miR-375	Song et al. * (34)	UBE3A, SP1, role in EMT
miR-378a	Fekete et al. (24)	/
miR-378c	Fekete et al. (24)	/
miR-378d-2	Fekete et al. (24)	/
miR-411	Wei et al. + (30)	STK38L, STK17A
miR-492	Liu et al. (25)	ADAMTS1, CD44, TIMP2, MZF-1, CD147, PTEN, SOX7
miR-502	Fekete et al. (24)	/
miR-5586	Fekete et al. (24)	FOS, GNB1, CREB1, GNAQ, GRIN2A, GRIN2B, FOS, GSK3B, PPARGC1A, FOXO1
miR-605	Sun et al. (36)	MDM2
miR-7702	Fekete et al. (24)	/
**Circulating miRNAs**		
**miRNA ID**	**Reference describing the miRNA**	**Potential targets of miRNAs**
miR-145	Wei et al. (32)	HLTF
miR-375	Song et al. * (34)	UBE3A, SP1, role in EMT
miR-411	Wei et al. + (30)	STK38L, STK17A

EMT, epithelial–mesenchymal transition**; +** and * indicate the same study in tissue and circulating miRNAs.

**Table 3:** Quality assessment of the studies included in the present review.

Criteria *	Fekete et al. (24)	Liu et al. (25)	Pedroza-Torres et al. (26)	Fan et al. (27)	Chen et al. (28)	Ke et al. (29)	Gao et al. (31)	Wei et al. (30)	Wei et al. (32)	Liu et al. (33)	Song et al. (34)	Chen et al. (35)	Sun et al. (36)
1.													
2.													
3.	NA	NA	NA	NA	NA	NA	NA	NA	NA	NA	NA	NA	NA
4.													
5.													
6.	NA	NA	NA	NA	NA	NA	NA	NA	NA	NA	NA		NA
7.	NA	NA	NA	NA	NA	NA	NA	NA	NA	NA	NA	NA	NA
8.	NA	NA	NA	NA	NA	NA	NA	NA	NA	NA	NA	NA	NA
9.													
10.													
11.													
12.	NA	NA	NA	NA	NA	NA	NA	NA	NA	NA	NA	NA	NA
13.	NA	NA	NA	NA	NA	NA	NA	NA	NA	NA	NA	NA	NA
14.													

*Criteria: 1) Was the research question or objective in this paper clearly stated? 2) Was the study population clearly specified and defined? 3) Was the participation rate of eligible persons at least 50%? 4) Were all the subjects selected or recruited from the same or similar populations (including the same time period)? Were inclusion and exclusion criteria for being in the study prespecified and applied uniformly to all participants? Including period and place of recruitment (setting and geographic location) adequately described 5) Were a sample size justification, power description, or variance and effect estimates provided? 6) For the analyses in this paper, were the exposure(s) of interest measured prior to the outcome(s) being measured? 7) Was the time frame sufficient so that one could reasonably expect to observe an association between exposure and outcome if it existed? 8) For exposures that can vary in amount or level, did the study examine different levels of the exposure as related to the outcome (e.g., categories of exposure or exposure measured as a continuous variable)? 9) Were the exposure measures (independent variables) clearly defined, valid, reliable, and implemented consistently across all study participants? (Assessment of miRNA analysis and validation in an independent cohort) 10) Was the exposure(s) assessed more than once over time? 11) Were the outcome measures (dependent variables) clearly defined, valid, reliable, and implemented consistently across all study participants? (Assessment of response) 12) Were the outcome assessors blinded to the exposure status of participants? 13) Was loss to follow-up after baseline 20% or less? 14) Were key potential confounding variables measured and adjusted statistically for their impact on the relationship between exposure(s) and outcome(s)? Highlighted in red criteria adapted to the papers analyzed.

Quality was rated as poor (0–4 out of 14 questions), fair (5–10 out of 14 questions), or good (11–14 out of 14 questions).

Green, yes; Red, no; Orange, partial (i.e., validation on the same cohort but with different technique); NA, not applicable.

### miRNA Expression in Cervical Cancer and Chemotherapy and Radiotherapy

Among the six papers, five included profiling of miRNAs, whereas one analyzed single miRNAs based on literature evidence. In particular, four papers investigated miRNAs through microarray or Taqman array, while one paper used Genomic Data Commons (GDC) data portal (https://portal.gdc.cancer.gov/) to retrieve and analyze miRNAseq data on CC patients under treatment. The first miRNA profiling in CC dates back to 2013, when Ke et al. (29) investigated SCC frozen tumor samples from 18 patients, of which 7 were resistant and 11 were sensitive to radiotherapy in association with cisplatin; therapeutic resistance or sensitivity was defined based on histological findings on cervical biopsies that were sampled 6 months after completion of radiotherapy. Eight miRNAs were significantly deregulated (miR-16–2*, miR-18a, miR-21, miR-23a, miR-30*, miR-181a, miR-221, and miR-378), and 6 were selected to be further validated in cell models, showing that miR-181a had the most important role in CC radiosensitivity. The same study cohort was used by Chen et al. (28) to further investigate the role of miR-181a. However, in this case, the main goal was to understand the contribution of miR-181a in platinum therapy rather than radiotherapy. To do that, the authors carried out *in vitro* and *in vivo* experiments and corroborated that miR-181a acts as an oncogene to enhance the chemoresistance through the pro-apoptotic protein kinase PRKCD. A second miRNA profiling was carried out by Fan et al. (27) with the aim of shedding light on the relationship between miRNAs and paclitaxel sensitivity. The work started by analyzing the miRNA expression profiles in two CC cell lines and their paclitaxel-resistant counterparts; 18 deregulated miRNAs were detected in paclitaxel-resistant cells compared with paclitaxel-sensitive cells, and 6 of those were randomly selected to be further tested by real-time PCR (RT-PCR). The results were consistent with the array results, and miR-125a was the most deregulated miRNA, with a significant downregulation in resistant cells. After careful *in vitro* and *in vivo* analyses and the identification of Signal transducer and activator of transcription 3 (STAT3) as a potential miR-125a target, the authors evaluated miR-125a in 43 CC tissue samples that were collected before any type of treatment. The Response Evaluation Criteria in Solid Tumors (RECIST) (38) were adopted to assess the effect of chemotherapy on progression-free survival (PFS) and overall survival (OS), and CC patients were grouped based on high or low miR-125a expression. Based on that, low miR-125a expression was significantly correlated with poorer PFS, OS, and response rate compared with the high miR-125a expression group. Moreover, miR-125a expression was significantly downregulated in non-response patients. In the same way, Pedroza-Torres et al. (26) analyzed a total of 41 CC samples. Specifically, 10 of those were used for miRNA profiling, while 31 samples represented the validation cohort. Therapeutic responses were evaluated through the RECIST criteria and computed axial tomography scans, and patients were classified as having a complete response (CR) in case of disappearance of all signs of cancer in response to treatment or having no response (NR) if showing partial, progressive, or stable disease. The miRNA profiling on the discovery set displayed 101 differentially expressed miRNAs between the 5 CR and 5 NR patients. A subset of 7 miRNAs (miR-31-3p, miR-3676, miR-125a-5p, miR-100-5p, miR-125b-5p, miR-200a-5p, and miR-342) was assessed in the independent group of 31 samples by single-miRNA assay showing consistency with the global profile. Moreover, CC patients were dichotomized into two groups (i.e., low and high expression levels), and disease-free survival (DFS) was assessed showing that low expression was a significant predictor of non-response to standard treatment. Similarly, Liu et al. (25) performed miRNA profiling on a small study cohort of 6 CC patients, of whom 3 were resistant and 3 were sensitive to concomitant chemoradiotherapy. In this work, patients with recurrent disease within 12 months after completion of first-line therapy were defined resistant, while the ones with no recurrence were termed sensitive. Twenty miRNAs showed a significant differential expression between the two sample groups, with miR-492 as the most deregulated. miR-492 was further validated in 104 CC samples, confirming a lower expression of miR-492 in treatment-resistant tumors. A higher miR-492 expression was also associated with pelvic lymph node metastasis (LNM), and *in vitro* experiments demonstrated that miR-492 overexpression promotes cell proliferation and migration and enhances the sensitivity of CC cells to irradiation by apoptosis.

A different approach was used by Fekete et al. (24), who retrieved miRNA expression data through the GDC data portal. The aim of the work was to identify miRNA predictive biomarkers in platinum-treated SCCs, regardless of the tumor site; for this reason, they included CC and lung and head and neck SCC (HNSC) for a total of 266 patients. Of the 94 CC patients, 16 were non-responders and 78 were responders, defined based on the presence of disease progression at 18 months. In the CC subgroup, 16 miRNAs that were differentially expressed between responder and non-responder patients were retrieved. Based on a miRNA similarity score, CC and HNSC were combined (for a total of 199 cases), and a logistic regression model including 6 miRNAs (miR-101-2, miR-632, miR-642a, miR-2355, miR-5586, miR-6728) was established; the model was generated by randomly dividing samples in the training set and the test set and was able to predict chemotherapy resistance with an area under the curve (AUC) of 0.897. Unfortunately, given the small sample size, the authors did not apply the model in the CC group alone and we cannot speculate on its performance in this specific type of tumor.

### miRNA Expression in Cervical Cancer and Radiotherapy

Five works evaluated miRNAs with regard to response to radiotherapy. None of these evaluated miRNAs by large profiling, but single-miRNA analysis was preferred. As mentioned, three studies analyzed circulating miRNAs, of which one in plasma and two conducted a parallel evaluation on tumor tissue and blood. In particular, in 2015, Song et al. (34) investigated a specific miRNA (miR-375) in both CC tumor and blood serum samples; in our knowledge, this was the first study to evaluate “liquid” miRNAs. In this case, the study cohort included 22 CC patients who were positive for high-risk (HR) HPV. miR-375, chosen based on previous literature evidence, showed lower levels in radioresistant patients compared with radiosensitive patients in both biological matrixes. Moreover, the role of miR-375 on radiosensitivity was further explored in cell line models. The results indicated a potential network between miR-375 and UBE3A, highlighting that miR-375 may promote radiosensitivity of HR HPV-positive patients, *via* p53 degradation. Similarly, Wei et al. (30) investigated miR-411 in 141 CC patients, in both CC tumor and blood samples. In this case, the cohort included 92 patients responding (complete and partial response) and 49 not responding (stable and progressive disease) to radiotherapy (30). miR-411 was increased in the radioresponsive group vs. the non-responsive patients, regardless of the type of sample (i.e., blood or tissue). Receiver operating characteristic (ROC) curves were used to assess the predictive value of miR-411 for radiotherapy efficacy, suggesting that miR-411 had good predictive value in tissues and peripheral blood in CC. Moreover, miR-411 was significantly higher in patients with longer 3-year OS and PFS rates compared with those with a lower miR-411 expression. Another interesting work is presented by Wei et al. who performed an evaluation of plasmatic miR-145 on 120 CC patients as a potential biomarker of the radiotherapy response (32). Indeed, from a previous report (39), a correlation between low levels of miR-145 in CC tissues and lymph node metastases and advanced clinical stage was observed, but its correlation with radiotherapy response had not been investigated. Among the 120 CCs, of which 68 were complete and 52 were incomplete responders, patients achieving a complete response presented higher levels of plasmatic miR-145 compared with the others; even in this case, ROC analysis confirmed the predictive value of miR-145 in differentiating complete from incomplete responders. Unfortunately, no validations of these interesting results in independent cohorts neither in cell models were carried out.

Two additional papers focused on tumor tissue miRNAs and radiotherapy response were also retrieved in our literature analysis (31, 33). Specifically, the one from Liu et al. (33) explored the role of miR-18a in regulating the radiosensitivity in CC. Indeed, the involvement of miR-18a has been reported in several cancer types, including but not limited to bladder cancer, hepatocellular carcinoma, and colon cancer (40–42), but its role in CC was unknown. The expression of miR-18a was investigated in 48 CC samples showing that it was significantly higher in radiosensitive patients compared with radioresistant patients.

Gao et al. (31) aimed to evaluate the role of a long non-coding RNA (lncRNA), GAS5, and miR-106 in CC. GAS5 is a known tumor suppressor that acts as a sponge of miR-106b. The analysis was performed on 20 CC samples of which 11 were from radiosensitive and 9 were from radioresistant patients; the RT-PCR analysis highlighted that GAS5 levels were significantly decreased, while miR-106b expression was increased in radioresistant tissues compared with radiosensitive tissues. Further *in vitro* studies from the same authors allowed to establish that miR-106b negatively regulated Immediate Early Response 3 (IER3), an important player in modulating sensitivity to chemotherapeutic drugs.

### miRNA Expression in Cervical Cancer and Neoadjuvant Treatment

Two papers explored miRNA levels in relation to the efficacy of NAC before radical hysterectomy in comparison with patients not treated before resection. The goal of the works was to assess the efficacy of NAC rather than to evaluate miRNAs. Sun et al. (36) analyzed miR-34 and miR-605 in 21 CC patients, of whom 11 were treated with the neoadjuvant protocol. miR-34 and miR-605 were chosen due to their belonging to two protein networks (p53-miR34-E2F1 and p53-miR-605-Mdm2) related to aggressive oncogenic signaling cascades in different tumors. The specimens were collected during surgery, and miRNA levels were analyzed. Both miR-34a and miR-605 were higher in patients treated with NAC compared with the non-treated ones. As mentioned, this paper aimed to evaluate NAC efficacy, so the authors reported a smaller tumor size in NAC patients and lower metastasis rate and better DFS and OS. However, the value of miRNAs in discriminating responsive vs. non-responsive patients was not evaluated, and the paper evaluated only the difference in miRNA levels at the time of surgery between NAC-treated and non-treated patients.

In the same way, Chen et al. (35) intended to analyze miR-143 in relation to NAC. miR-143 was selected because literature reported its involvement in CC (43, 44). The total cohort included 77 CC samples; however, only 34 patients were treated with NAC. For each patient, the tumor material was collected before and after NAC and a comparison in terms of miR-143 levels was carried out in 24 cases. No significant differences at the two time points were recorded, suggesting that miR-143 does not contribute to mediate taxol sensitivity. However, this study is of particular interest because it is the sole to compare miRNA levels before and after treatment on the same patients, allowing to evaluate NAC effects on a selected miRNA. Even in this case, the type of response (i.e., responsive or not) was not considered (35).

## Discussion

CC is one of the most common cancer types among women of developing countries, being the fourth most common female cancer worldwide (45).

While screening programs and HPV vaccines have led to a reduction of CC in developed countries, in developing countries CC remains an important issue, with ~80%−90% of patients at stages III–IV (36, 45).

Concomitant chemoradiotherapy as definitive approach remains the gold standard for locally advanced tumors, while surgery alone, or followed by radiotherapy, is the standard for early stages; NAC is offered to patients who wish to reduce cancer before surgical intervention, but this clinical approach is not considered a standard therapy. Unfortunately, a certain percentage of patients do not respond to the therapeutic plan with poor prognosis, and the prediction of response represents an important clinical issue. In this context, the identification of predictive biomarkers of chemotherapy and radiation sensitivity denotes an unmet clinical need.

In the last decade, the clinical value of miRNAs has been widely explored in cancer due to their recognized role in tumor development, progression, and response to therapy. Increasing evidence has shown their importance in mediating several biological processes in CC, while the number of reports investigating miRNAs in the therapeutic response is limited; this appears to be particularly relevant if compared with other cancers, including but not limited to ovarian, lung, or breast cancer (46–48), where the literature body is quickly increasing. In the present review, we aimed to provide an overview of the current literature on tumor tissue and circulating miRNAs significantly associated with therapeutic response in CC. In our analysis, we retrieved only 13 papers falling in our scope, of which 6 works evaluated miRNAs in patients treated with both chemotherapy and radiotherapy and 5 in radioresistant or non-resistant CC patients. In general, the analyses were heterogeneous in terms of type of miRNAs (i.e., tumor or circulating miRNAs) and techniques. In particular, 3 out of 13 papers analyzed circulating miRNAs, however one in peripheral blood, one in plasma, and one in serum; four of 13 used large profilings to simultaneously screen multiple miRNAs, one retrieved the miRNA levels from an available omics database, whereas the remaining papers adopted RT-PCR as the main technique to evaluate a limited number of miRNAs. Another source of heterogeneity was related to the assessment of therapeutic response. As summarized in **Table 1**, in a few cases, the cut-off to judge the responsiveness was 6 months, while in other cases, it was 12 or 18 months. Six works analyzed HPV together with miRNAs, as it is recognized as a risk factor for CC development, although the remaining works did not mention that.

Overall, as previously mentioned, the studies on miRNAs and therapeutic response available in the literature can be grossly divided in three groups according to the type of therapeutic plan (i.e., concomitant chemoradiotherapy and radiotherapy or radiotherapy alone as adjuvant setting or NAC); however, even considering this aspect, the consensus among the studies appears very limited; **Table 2** offers a good perspective of that, showing the large number of significant miRNAs but the concomitant lack of agreement. Moreover, our review highlights a wide range of treatments reported in CC patients that would require a global centralization to provide uniformity of care, at least in high-income countries where the disease is rarer. With all of these considerations in mind, it is comprehensible that no clinical translation has happened yet, and further research will be needed to outline reliable miRNA candidate biomarkers. In addition, the lack of standardized protocols, including sample collection, RNA extraction, and techniques, as well as definition of therapeutic response assessment, hampers the comparison of results between independent studies. On the other hand, identification of one or a few miRNAs able, by themselves, to accurately discriminate responsive/non-responsive patients seems unrealistic, while the best approach would be combining multiple variables (including, but not limited to miRNAs and clinical parameters). Another interesting observation arises from the limited research on circulating miRNAs. Indeed, if the number of works adopting this type of analysis is limited to three studies comparing responsive vs. non-responsive patients, “liquid” miRNAs have not been employed to monitor the response over specific treatment with a wide knowledge gap to fill. As a consequence, the research on circulating miRNAs in CC is still in its embryonal phase, and no reliable miRNA candidates to accurately follow the treatment response “in real time” have been explored, leaving space for additional studies.

In conclusion, to the best of our knowledge, this is the first systematic revision dealing with the role of miRNAs in the therapeutic response in CC. The potential application of miRNAs in CC remains to be elucidated given the inconsistent conclusions reported by different studies. This could be in part due to the limited number of investigations, the small sample size, the lack of standardized protocols to appropriately assess the miRNA contribution, and the heterogeneity of therapeutic schemes.

Further studies with standardized procedures and larger cohorts of patients should be warranted to foster the identification of miRNAs of potential clinical significance in CC.

## Data Availability Statement

The original contributions presented in the study are included in the article/supplementary material. Further inquiries can be directed to the corresponding authors.

## Author Contributions

Conceptualization: GR and AMP. Data curation: GR and FG. Writing—original draft preparation: GR, FG, GD, MT, EC, AM, and AP. Writing—review and editing: GR, FG, GD, MT, AM, PI, PH, SA, and AMP. All authors have read and agreed to the published version of the article.

## Funding

The work was supported by the Fondazione Cassa di Risparmio in Bologna (Carisbo) project number #19094 to AMP.

## Conflict of Interest

The authors declare that the research was conducted in the absence of any commercial or financial relationships that could be construed as a potential conflict of interest.

## Publisher’s Note

All claims expressed in this article are solely those of the authors and do not necessarily represent those of their affiliated organizations, or those of the publisher, the editors and the reviewers. Any product that may be evaluated in this article, or claim that may be made by its manufacturer, is not guaranteed or endorsed by the publisher.
